# Establishing the Mutational Spectrum of Hungarian Patients with Familial Hypercholesterolemia

**DOI:** 10.3390/genes13010153

**Published:** 2022-01-15

**Authors:** László Madar, Lilla Juhász, Zsuzsanna Szűcs, Lóránt Kerkovits, Mariann Harangi, István Balogh

**Affiliations:** 1 Division of Clinical Genetics, Department of Laboratory Medicine, Faculty of Medicine, University of Debrecen, 4032 Debrecen, Hungary; madar.laszlo@med.unideb.hu (L.M.); szucs.zsuzsanna@med.unideb.hu (Z.S.); 2 Doctoral School of Molecular Cell and Immune Biology, University of Debrecen, 4032 Debrecen, Hungary; 3 Division of Metabolic Diseases, Department of Internal Medicine, Faculty of Medicine, University of Debrecen, 4032 Debrecen, Hungary; lilla.juhasz@hotmail.com (L.J.); harangi@belklinika.com (M.H.); 4 Department of Nephrology, South-Budapest Center Hospital St. Imre Teaching Hospital, 1115 Budapest, Hungary; kerkovits.lorand@gmail.com; 5 Department of Human Genetics, Faculty of Medicine, University of Debrecen, 4032 Debrecen, Hungary

**Keywords:** familial hypercholesterolemia, FH, Hungary, *LDLR*, *APOB*, next-generation sequencing, NGS

## Abstract

Familial hypercholesterolemia (FH) is one of the most common autosomal, dominantly inherited diseases affecting cholesterol metabolism, which, in the absence of treatment, leads to the development of cardiovascular complications. The disease is still underdiagnosed, even though an early diagnosis would be of great importance for the patient to receive proper treatment and to prevent further complications. No studies are available describing the genetic background of Hungarian FH patients. In this work, we present the clinical and molecular data of 44 unrelated individuals with suspected FH. Sequencing of five FH-causing genes (*LDLR*, *APOB*, *PCSK9*, *LDLRAP1* and *STAP1*) has been performed by next-generation sequencing (NGS). In cases where a copy number variation (CNV) has been detected by NGS, confirmation by multiplex ligation-dependent probe amplification (MLPA) has also been performed. We identified 47 causal or potentially causal (including variants of uncertain significance) *LDLR* and *APOB* variants in 44 index patients. The most common variant in the *APOB* gene was the c.10580G>A p.(Arg3527Gln) missense alteration, this being in accordance with literature data. Several missense variants in the *LDLR* gene were detected in more than one index patient. *LDLR* variants in the Hungarian population largely overlap with variants detected in neighboring countries.

## 1. Introduction

Familial hypercholesterolemia (FH, MIM#143890) is one of the most common autosomal, dominantly inherited diseases; it is a disorder of the cholesterol metabolism and one of the major risk factors for early arteriosclerosis and cardiovascular complications. Disease prevalence is 1:200–250. The estimated FH prevalence in Hungary was found to be 1:340 [1]. Early detection of the disease and proper treatment could greatly improve the clinical outcome. The diagnosis of FH is primarily clinical, and it is still widely underdiagnosed and undertreated. To aid diagnosis, a FH score calculation system has been set up based on the recommendations of the European Society of Atherosclerosis [2]. This scoring system is known as the Dutch Lipid Clinic Network (DLCN), based on which it can be decided if the patient’s diagnosis of FH is unlikely, possible, probable, or definite. In most cases, pathogenic variants of the *LDLR* and *APOB* genes are responsible for familial hypercholesterolemia, and sometimes alterations in the *PCSK9* gene, but more FH-related genes have also been described, such as the *LDLRAP1* and *STAP1* genes. Variants in these genes have a negative effect on the proper elimination of low-density lipoproteins (LDL) from the blood, which results in an elevated LDL cholesterol (LDL-C) and total cholesterol (TC) level.

In the case of a heterozygous alteration, the FH patient has an LDL-C level higher than 5 mmol/L, whereas homozygous or compound heterozygous alterations generally result in an LDL-C level higher than 13 mmol/L [3]. The clinical symptoms are more severe in patients with homozygous or compound heterozygous variants. If phenotypes such as cutaneous and tuberous xanthomas, corneal arcus, or atherosclerotic cardiovascular disease (ACVD) are present—generally under the age of 20—this raises the suspicion of a homozygous variant in one of the FH-causing genes.

To date, over 2100 *LDLR* and 250 *APOB* pathogenic variants have been described in the literature (Professional Human Gene Mutation Database, 2021.3 release). No mutational hotspots have been identified in the *LDLR* gene; the genetic variants are spread from the promoter region to the very last exon of the gene. According to literature data, pathogenic variants occur more frequently in some regions of the gene, such as the ligand-binding domain and the epidermal growth factor-like domain.

### 1.1. Structure and Function of ApoB

The *APOB* gene is located on chromosome 2, consists of 29 exons and encodes 4563 amino acids. Apolipoproteins play an important role in binding the lipoprotein particle to lipoprotein receptors. One of the main roles of lipoproteins is supplying the cells with lipids and transporting the unnecessary lipids. ApoB has two forms, ApoB 100 and ApoB 48. Both forms are the product of the *APOB* gene, but the ApoB 48 is a truncated version of ApoB 100. ApoB 100 is produced in the liver, whereas ApoB 48 is produced in the proximal small intestines.

### 1.2. Structure and Function of LDLR

The human LDLR is encoded by a gene of ~44.5 kb located on chromosome 19p13.3. The 18 exons of the gene are translated into an 860 amino acid-long type I transmembrane protein. The *LDLR* gene has five functional domains: an N-terminal ligand-binding region, an epidermal growth factor (EGF)-precursor homology region, a region containing O-linked sugars, a transmembrane domain, and a C-terminal cytosolic domain.

The LDLR mediates the clearance of cholesterol and cholesteryl ester-containing low-density lipoprotein (LDL) particles from blood [4]. LDLR plays an important role in cholesterol homeostasis, and this is well illustrated by the fact that more than 2000 *LDLR* variants have been found in patients with familial hypercholesterolemia (FH) that can impair LDLR activity at different levels and therefore are classified according to their phenotypic behavior as follows: class 1 (no protein synthesis), class 2 (partial or complete retention of LDLR in the endoplasmic reticulum, class 3 (defective binding to apoB, class 4 (defective endocytosis) and class 5 (diminished LDLR recycling capacity) [5].

Our goal was to examine the genetic background of patients with familial hypercholesterolemia in Hungary to map variants in the Hungarian population, as no such data have been available to date. Here, we describe the clinical and molecular data of 44 unrelated suspected FH patients.

## 2. Patients and Methods

### 2.1. Patients

Patients clinically diagnosed with FH were referred to our laboratory for genetic testing from all around Hungary. Family members of index patients who tested positive were also examined when available. All participants or their legal representatives gave informed consent to genetic testing according to national regulations. Relevant clinical data were available in the case of most patients.

Clinical diagnosis, whenever possible, was established by a clinical geneticist or internist based on the patient’s laboratory findings and possible phenotypic symptoms. Among the patients with a pathogenic alteration, there were 31 female and 13 male patients. The mean age of the patients was 48 years (14–78 years). The average LDL-cholesterol level was 7.2 (mmol/L) and the average total cholesterol level was 10.4 (mmol/L) in those who had at least one FH-causing variant.

### 2.2. Methods

#### 2.2.1. Isolation of Genomic DNA

Genomic DNA was isolated from peripheral blood leukocytes using the QIAamp Blood Mini kit (Qiagen GmbH, Hilden, Germany).

#### 2.2.2. Sequencing of Genes Related to Familial Hypercholesterolemia

Genes related to familial hypercholesterolemia were analyzed using the Devyser FH v2 kit (Devyser AB, Hägersten, Sweden). This amplicon-based library preparation kit can examine the coding region and exon/intron boundaries (minimum +/− 10 base pairs) of the following genes: *APOB*: NM_000384.3, *LDLR*: NM_000527.5, *PCSK9*: NM_174936.4, *LDLRAP1*: NM_015627.3, *STAP1*: NM_012108.4.

Sequencing was performed on Illumina MiSeq with 300 cycles according to the manufacturer’s protocol. Raw data analysis was carried out with the NextGENe (SoftGenetics, State College, PA) software, with a minimum coverage requirement of 40x. Copy number variation detection was performed based on coverage data.

The detected pathogenic variants were confirmed by Sanger sequencing. Primers were designed using Primer3 software (https://primer3.ut.ee/) (accessed on 15 October 2021). Primers are listed in Supplementary Table S1.

#### 2.2.3. MLPA

Confirmation of a CNV detected by NGS was performed in three cases by MLPA method using SALSA MLPA Probemix P062 Mix 1 (MRC Holland, Amsterdam, The Netherlands) according to the manufacturer’s protocol.

#### 2.2.4. Variant Filtering and Interpretation

All detected variants with an MAF > 0.01 (minor allele frequency) in the gnomAD population database were filtered. The remaining variants were classified according to the ACMG standards and guidelines [6]. A web-based interpretation tool, Franklin (Genoox), was used to assist the classification [7]. HGMD Professional and ClinVar databases were also used in variant interpretation.

#### 2.2.5. Breakpoint Determination

Primers were designed for intron 6 and intron 8 in the *LDLR* gene surrounding the deletion to determine the suspected breakpoints (Supplementary Table S1). Following amplification, Sanger sequencing was performed.

## 3. Results

We identified a total of 47 variants related to familial hypercholesterolemia in 44 unrelated Hungarian index patients with suspected FH. The variants detected are summarized in Table 1, Table 2, Table 3 and Table 4. The majority of the detected variants (40 alterations) was located in the *LDLR* gene, whereas seven cases were in the *APOB* gene. No pathogenic variants related to FH were detected in the *PCSK9, LDLRAP1* or *STAP1* genes.

**Figure 1:**
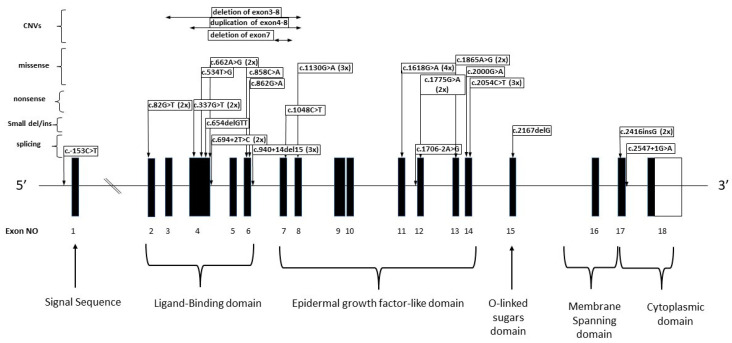
This figure shows the detected *LDLR* variants and their localization in the gene.

Regarding the distribution of the variants throughout the *LDLR* gene, 1 variant was located in the promoter region, 17 in the ligand-binding domain, 18 in the epidermal growth factor (EGF) precursor homology domain, one in the O-linked sugar region, and a further 3 variants were found in the cytoplasmic domain (Figure 1).

### 3.1. APOB Alterations

We detected a pathogenic alteration in the *APOB* gene in heterozygous form in seven patients. We identified the most frequent FH-causing *APOB* variant c.10580G>A, p.(Arg3527Gln) [8] in four patients.

In two index patients, we detected two missense variants (c.8213T>A, p.(Ile2738Lys), c.10438A>G, p.(Lys3480Glu)) where pathogenicity is unclear, but they are missing from population databases, therefore not present in the normal population. Furthermore, we did not detect any other alteration in the genes examined in either of the patients. According to the ACMG classification criteria, they are classified as variants of uncertain significance (VUS), so further testing is needed to decide their pathogenicity.

In exon 29 of the *APOB* gene, the c.13242delG frameshift variant was detected. This deletion results in a premature stop codon at the 4415 amino acid position, leading to a truncated protein. As the variant is located in the last exon, it is unlikely that the consequence of it would be nonsense-mediated mRNA decay, rather, the truncated protein likely lacks 148 amino acids from its C-terminal. The variant is not present in the population databases, but ClinVar describes it as a variant of uncertain significance with a single submission of a clinical test result (ClinVar: SCV001357780). The prediction algorithms classify it as VUS/Likely Pathogenic. Downstream to this variant, other pathogenic truncating alterations have been described, which also suggests that this variant is a pathogenic one [28]. Table 1 presents the *APOB* variants in details.

### 3.2. LDLR Alterations

We detected 40 alterations in the *LDLR* gene in a total of 37, patients of which three patients carried variants in the compound heterozygous form (i.e., phenotypically homozygous).

#### 3.2.1. Patients with Missense LDLR Alterations

Several missense variants detected in the *LDLR* gene were present in more than one index patient. The most common variant, c.1618G>A, p.(Ala540Thr), was present in four families, the c.1130G>A, p.(Cys377Tyr) change in three families, and the c.2054C>T, p.(Pro685Leu) was also detected in three families. The c.662A>G, p.(Asp221Gly), c.1775G>A, p.(Gly592Glu), c.2000G>A, p.(Cys667Tyr) and c.1865A>G, p.(Asp622Gly) alterations were detected in 2-2 families (Table 2).

#### 3.2.2. Patients with Nonsense Variants

The c.82G>T, p.(Glu28*) and c.337G>T, p.(Glu113*) nonsense variants were detected in 22 families, whereas the c.1048C>T, p.(Arg350*) alteration was present in one family in heterozygous form. In patient 13, the c.337G>T, p.(Glu113*) variant was detected together with another pathogenic missense change (Table 2).

#### 3.2.3. Patients with Splicing Alterations

We detected variants resulting in a splicing defect in eight patients (Table 2). Two of these variants were detected in more than one index patient. The c.940_940+14del was detected in three patients (patients 8, 11, and 23) as the causal variant. This variant is likely to affect protein splicing and damages protein function. We found the c.694+2 T>C variant in two families.

A previously unreported splicing variant of unknown pathogenicity, c.1706-2A>G, was detected in the *LDLR* gene in one patient (patient 34) (Table 2.).

#### 3.2.4. Small Duplications/Deletions in the LDLR Gene

The c.2416dupG, p.(Val806Glyfs*11) variant was detected in two families in a heterozygous form. This is a 1 bp insertion in exon 17 of the *LDLR* gene in a homopolymer region consisting of five guanines. As a result of the insertion, there will be six guanines instead of five, which disrupts the reading frame, leading to a premature stop codon. The variant belongs to class three as it results in a defective receptor.

We detected the c.2167delG, p.(Glu723Argfs*7) alteration in patient 28 in a heterozygous form. This small deletion in exon 15 of the *LDLR* gene disrupts the reading frame and leads to a premature stop codon. In this patient, we also detected a second heterozygous missense change, the c.862G>A, p.(Glu288Lys).

An in-frame deletion (c.654_656delTGG, p.(Gly219del)), was detected in a single patient in our cohort (Table 2).

#### 3.2.5. Patients with Compound Heterozygous *LDLR* Alterations

We had three patients in our cohort with a compound heterozygote genotype (P12, P13, P28). The clinical symptoms were severe in all three patients, as can be seen in Table 3.

**Figure 2:**
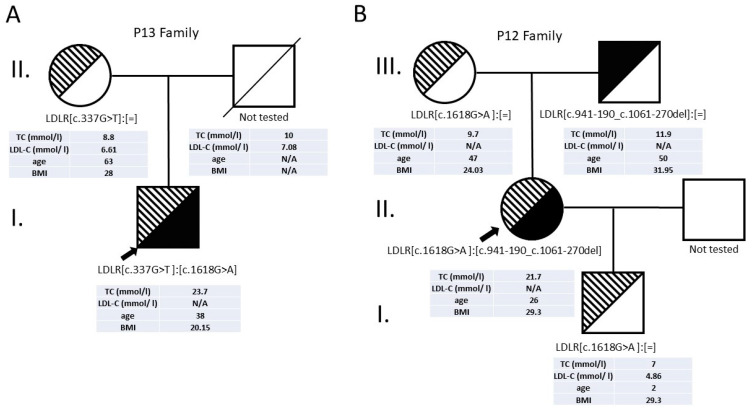
Pedigree of patients 13 and 12. (**A**) Patient 13 family members. The father of the proband could not be tested. (**B**) Patient 12 family members. Three generations were tested.

Patient 13 had a nonsense (c.337G>T, p.(Glu113*)) and a missense (c.1618G>A, p.(Ala540Thr)) variant in a heterozygous form. Cascade screening revealed that the patient inherited the nonsense variant from the mother. The father had previously died because of a cardiovascular complication, so the paternal DNA sample was not available for testing (Figure 2).

In Patient 28, we detected a missense (c.862G>A, p(Glu288Lys)) and a frameshift (c.2167delG, p.(Glu723Argfs*7)) heterozygous variant.

The c.2167delG variant was described previously, where a patient had highly elevated TC level [18]. Our patient also had significantly elevated LDL-C and TC levels. Although xanthoma has not been described in this case, due to his young age (13 years), it could develop later. DNA samples of family members of patient 28 were not available for testing.

**Figure 3:**
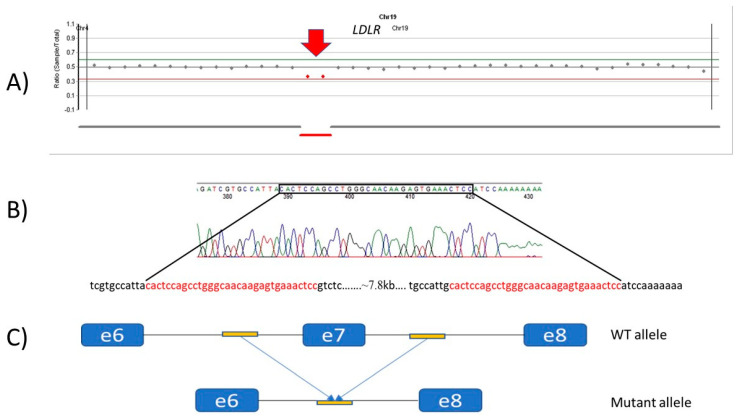
Breakpoint determination of the deletion of exon 7. (**A**) Coverage-based CNV from NGS data. The red arrow indicates the complete heterozygous deletion of exon 7 covered by 2 amplicons (Patient 12). (**B**) The result of Sanger sequencing of the mutant allele amplified by the PCR reaction. The short homologue DNA sequence is highlighted in the frame and highlighted in red. (**C**) The wild-type and mutant allele without exon 7. The mutant allele contains only one copy of a short homologue DNA sequence.

Patient 12 has a missense change (c.1618G>A, p.(Ala540Thr)) and deletion of the whole exon 7 (c.941-190_c.1061-270del). We performed targeted testing of the available family members as well, which proved that the two variants were inherited in trans (Figure 2). We have successfully determined the breakpoints of this deletion in exon 7 of the *LDLR* gene. Only one of the two short 32 bp-long homologue DNA sequences before and after exon 7 was detected in the PCR product. The copy loss of this sequence indicates that deletion of exon 7 occurred (Figure 3).

#### 3.2.6. Patients with CNV *LDLR* Variants

We detected a copy number variation in the *LDLR* gene in three patients, in one case in a compound heterozygous form in the above-described patient 12.

**Figure 4:**
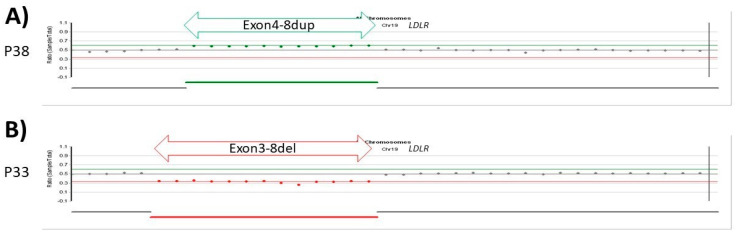
Coverage-based CNV detection in the *LDLR* gene. (**A**) In patient 38, the duplication of exons 4–8 was detected based on coverage data. The green arrow indicates the location and size of the duplication. (**B**) In patient 33, the deletion of exons 3–8 was detected based on coverage data. The red arrow indicates the location and size of the deletion.

In patient 38, we detected the duplication of exons 4–8 and in patient 33 we detected the deletion of exons 3–8 in a heterozygous form based on coverage from NGS data (Figure 4 and Table 4).

## 4. Discussion

Patients clinically diagnosed with FH were examined with a next-generation sequencing method that detects not only the small-scale variants but large-scale deletions and insertions as well.

One of the *LDLR* alterations (c.1706-2A>G) is described here the first time, whereas the other 39 *LDLR* variants have already been reported in the literature. Although the deletion of exon 7 of the *LDLR* gene has been described previously [29], we have successfully determined the breakpoints as well. We detected a familial hypercholesterolemia-related variant in a total of 59 individuals during the examination of our cohort, of which 44 index patients and 15 family members had a variant classified as pathogenic/likely pathogenic according to the ACMG criteria, and in two cases the alteration was a variant of unknown significance (Supplementary Table S2). Regarding the distribution of the *LDLR* variants, most of them are in the ligand-binding domain and the epidermal growth factor-like domain.

To our knowledge, no study describing the variants related to familial hypercholesterolemia in the Hungarian population has been published before. Regarding the mutation spectrum, there is a large overlap between Hungary and neighboring countries, such as Poland or the Czech Republic. The most common variants in the *LDLR* gene in the Polish population can be found in our cohort as well [21,26].

The 1618G>A, p.(Ala540Thr) missense change was detected in four index patients (21,42,12,13), and in a further two family members during cascade testing. This pathogenic variant has been described in several European and Latin American populations, and it is a commonly occurring alteration among our patients as well. The phenotypes of our patients are similar to the ones described in the literature. This variant is located in the epidermal growth factor (EGF)-precursor homology domain. Its pathogenicity is demonstrated by the fact that, as described in the literature before, patients homozygous for this variant present with severe clinical symptoms at a young age such as premature coronary heart disease, tendon xanthomas and high total cholesterol level [19,30].

The c.1130G>A, p.(Cys377Tyr) missense change was detected in three families. First, it was described in a study examining Swedish FH patients [17]. Its pathogenicity is further supported by the fact that other European cohort studies have also described this alteration in FH patients since then.

A missense change, c.2054C>T, p.(Pro685Leu) was detected in three families in our cohort. According to a comprehensive study describing more than 2000 FH-causing variants, this alteration is pathogenic. It should be noted that seven alterations were found in this study which have been described in several countries on every continent, and one of these is the c.2054C>T, p.(Pro685Leu) missense change [22]. This suggests the ancient nature of this variant.

The c.1775G>A, p.(Gly592Glu) missense change was detected in two patients in a heterozygous form. This variant has been previously described in several populations and was associated with a strong founder effect in a Greek region according to a previous study, because it was present in almost one-third of the patients examined and detected in a homozygous form in several cases. According to literature data, this class 5 variant has no drastic cholesterol-elevating effect associated with its homozygous form, suggesting that it results only in a small decrease in LDLR activity [20].

The nonsense alterations have been previously described as FH-causing pathogenic variants. These alterations result in a truncated form of the LDLR protein which is nonfunctional. The uptake of LDL particles is reduced in the liver and other tissues.

The c.-153C>T promoter variant has been previously detected in Czech FH patients. This variant is located in the conserved sterol-dependent regulatory element 2 (SRE2) in the promoter region of the *LDLR* gene. A luciferase-based functional assay has suggested that this variant may significantly reduce LDLR promoter activity [9].

The c.940_940+14del variant has been previously described in a study examining a Scandinavian population, where it segregated with the disease in the family tested [16]. In that study, both the clinical picture and the family study confirmed the pathogenicity of the alteration, and the development of premature cardiovascular disease has also been described in the patients carrying the variant. The LDL-C levels of our patients were also very high before the treatments (7.9–10.5 mmol/L).

Interestingly, the c.694+2 T>C variant was previously described as a founder variant in the Icelandic population that could be traced back to the 18th century. It affects a conservative splicing site and its pathogenicity has been proved by segregation studies as well [15].

The pathogenicity of the novel c.1706-2A>G variant is suggested by the fact that variants with other nucleotide changes have been previously identified in the same position (c.1706-2A>C; c.1706-2A>T). The c.1706-2A>T variant has been detected in two Arabic families, and further testing showed that the A>T exchange at this position is pathogenic because of the large reduction in LDLR mRNA levels [31].

The c.2416dupG, p.(Val806fs*11) variant was first described in a Swedish population study, then it appeared in Pakistani and other population studies as well. Elevated TC levels of the index patient and further eight family members were typical for heterozygous FH patients (7.76–12.93 mmol/L), whereas the 5-year-old child of the index patient had a TC level typical for homozygous FH patients (23.25 mmol/L) and had typical xanthoma [23]. Patient 18 from our cohort also had a significantly elevated TC level (8.1 mmol/L), coronary artery disease, and xanthelasma, whereas patient 44 had an elevated TC level (10 mmol/L).

The c.654_656delTGG, p.(Gly219del) deletion has been previously described in the Ashkenazi Jewish population as a founder variant, and identified as the FH-causing variant in more than 21% of the cases [13]. The variant results in the deletion of a glycine amino acid in the 219th position in the strongly conserved ligand-binding domain.

The c.337G>T, p.(Glu113*) variant is in exon 4 of the *LDLR* gene and it belongs to class 1 as it results in a null allele. It is a known pathogenic variant and was first described by Hobbs et al. [11]. The 1618G>A, p.(Ala540Thr) missense change is also a well-known pathogenic variant published in different studies examining different populations such as Spanish, German, Italian or Greek. It also appears in a previous study on genotype–phenotype relationships [30].

The c.862G>A, p.(Glu288Lys) missense change was first described in German patients [25]. In Italian patients, phenotypic consequences of the variant such as significantly elevated LDL-C and tendon xanthoma in several cases [14] were also shown.

Earlier, targeted *APOB* testing was characteristic because of the size of the *APOB* gene, examining the most common FH-causing variants as well [32]. Nowadays, due to the technological advancement of next-generation sequencing, the sequencing of the whole coding region and splice region of the *APOB* gene is available, therefore more FH and other lipid metabolism disorder-related alterations have been described [33].

In the *APOB* gene, the most common variant in the Hungarian population is the c.10580G>A p.(Arg3527Gln) missense change, which is in accordance with literature data. By testing the whole *APOB* coding region, we could detect a further three variants, of which two missense alterations have not been described before, and the third frameshift variant (c.13242delG, p.(Leu4415*)) has been previously reported in the ClinVar database, and is also related to FH. This result reflects on the fact that the sequencing of the whole *APOB* gene instead of targeted *APOB* testing can greatly improve the diagnostic sensitivity.

Our study revealed one novel variant in the *LDLR* gene, while most of the variants that are present in the Hungarian population have already been described.

The proportion of variant types and their localization within the gene are also highly similar in population studies.

Indicating the importance of establishing a genetic diagnosis, the timing of initiation of lipid-lowering therapies may greatly influence the onset and severity of expected complications. According to a large study that followed therapy in children for 20 years, early treatment greatly reduced or delayed the risk of cardiovascular complications in adulthood [34]. To initiate appropriate treatment, it is important to know whether the patient has a heterozygous, compound heterozygous or homozygous genotype. In addition, by cascade testing, the affected yet asymptomatic family members can be identified, leading to correct, timely treatment.

## Figures and Tables

**Table 1:** Clinical and phenotypic data in Hungarian patients with *APOB* variants and detailed summary of *APOB* variants.

Patient ID	Exon	Variant at cDNA Level	Variant at Protein Level	Variant Type	ACMG Classification	TC (mmol/L)	LDL-C (mmol/L)	Age	Gender	BMI	Dutch Score	Xanthoma, Xanthelasma	Reference
7	26	c.8213T>A	p.Ile2738Lys	missense	VUS	8.1	5.72	69	F	22.5	11	-	this study
4	26	c.10438A>G	p.Lys3480Glu	missense	VUS	8.0	5.3	65	F	25.5	13	-	this study
30	26	c.10580G>A	p.Arg3527Gln	missense	Likely Pathogenic	7.66	5.51	60	F	29.9	11	-	[8]
37	26	c.10580G>A	p.Arg3527Gln	missense	Likely Pathogenic	8.3	6.0	51	F	30.1	14	-	[8]
1	26	c.10580G>A	p.Arg3527Gln	missense	Likely Pathogenic	6.9	4.6	78	F	N/A	10	-	[8]
39	26	c.10580G>A	p.Arg3527Gln	missense	Likely Pathogenic	8.1	5.2	61	F	23.5	4	-	[8]
29	29	c.13242delG	p.Leu4415*	frameshift	VUS/Likely Pathogenic	5.62	7.3	51	M	26.3	16	-	SCV001357780

**Table 2:** Clinical and phenotypic data on Hungarian patients with *LDLR* variants and detailed summary of *LDLR* variants—patients with small-scale *LDLR* alterations.

Patient ID	Exon	Variant at cDNA Level	Variant at Protein Level	Variant Type	ACMG Classification	TC (mmol/L)	LDL-C (mmol/L)	Age	Gender	BMI	Dutch Score	Xanthoma, xanthelasma	Reference
43	promoter	c.-153C>T	p.?	point	VUS/Likely Pathogenic	9	6.13	21	F	22	13	-	[9]
10	2	c.82G>T	p.Glu28*	nonsense	Pathogenic	9.07	7.18	33	F	24.6	14	-	[10]
16	2	c.82G>T	p.Glu28*	nonsense	Pathogenic	13.3	10.23	53	F	34.7	18	-	[10]
35	4	c.337G>T	p.Glu113*	nonsense	Pathogenic	12.1	9.25	36	M	28.1	16	-	[11]
25	4	c.534T>G	p.Asp178Glu	missense	Pathogenic	4.2	2.4	48	M	27.8	11	-	[12]
41	4	c.654_656delTGG	p.Gly219del	in-frame deletion	Pathogenic	13	n/a	48	M	24.7	2	-	[13]
5	4	c.662A>G	p.Asp221Gly	missense	Pathogenic	5.6	3.8	66	F	25.2	2	-	[14]
20	4	c.662A>G	p.Asp221Gly	missense	Pathogenic	12	8.81	50	F	22	18	-	[14]
22	intron 4	c.694+2 T>C	p.?	splicing	Pathogenic	9.4	9.4	47	M	34.2	17	-	[15]
32	intron 4	c.694+2 T>C	p.?	splicing	Pathogenic	9	7.4	50	M	26.3	16	-	[15]
27	6	c.858C>A	p.Ser286Arg	missense	Pathogenic	9.2	6.93	34	F	22.9	13	-	[14]
8	6	c.940_940+14del	p.?	splicing	Pathogenic	10.4	7.9	30	F	25.8	14	-	[16]
11	6	c.940_940+14del	p.?	splicing	Pathogenic	10	10.5	14	F	22.4	17	-	[16]
23	6	c.940_940+14del	p.?	splicing	Pathogenic	11.4	8.7	53	F	22.2	17	-	[16]
9	7	c.1048C>T	p.Arg350*	nonsense	Pathogenic	11.9	9.75	52	F	n/a	16	n/a	[14]
36	8	c.1130G>A	p.Cys377Tyr	missense	Pathogenic	9.4	8.5	18	M	n/a	14	-	[17]
19	8	c.1130G>A	p.Cys377Tyr	missense	Pathogenic	8.4	6.2	18	F	19.6	12	-	[17]
24	8	c.1130G>A	p.Cys377Tyr	missense	Pathogenic	13.3	10.56	60	F	23	18	-	[17]
21	8	c.1618G>A	p.Ala540Thr	missense	Pathogenic	n/a	n/a	54	F	n/a	n/a	n/a	[18]
42	8	c.1618G>A	p.Ala540Thr	missense	Pathogenic	9.1	6.65	28	M	26.9	13	-	[19]
34	intron 11	c.1706-2A>G	p.?	splicing	Likely Pathogenic	8.2	5.8	50	F	30.5	12	-	this study
26	12	c.1775G>A	p.Gly592Glu	missense	Pathogenic	10.3	7.7	45	F	19.8	13	-	[20]
31	12	c.1775G>A	p.Gly592Glu	missense	Pathogenic	10.2	7.62	53	F	n/a	13	-	[20]
3	13	c.1865A>G	p.Asp622Gly	missense	Pathogenic	9.6	7.3	67	M	24.3	8	-	[21]
6	13	c.1865A>G	p.Asp622Gly	missense	Pathogenic	10	7.9	60	F	27.8	11	+	[21]
2	14	c.2000G>A	p.Cys667Tyr	missense	Pathogenic	12.9	10.4	64	F	30.1	10	-	[14]
17	14	c.2000G>A	p.Cys667Tyr	missense	Pathogenic	8.1	5.4	40	F	n/a	12	-	[14]
15	14	c.2054C>T	p.Pro685Leu	missense	Pathogenic	9.6	7.6	22	M	21.1	14	-	[22]
40	14	c.2054C>T	p.Pro685Leu	missense	Pathogenic	9.6	7.14	56	F	24.2	19	+	[22]
18	17	c.2416dupG	p.Val806fs*11	frameshift	Pathogenic	8.1	5.4	58	F	n/a	12	-	[23]
44	17	c.2416dupG	p.Val806fs*11	frameshift	Pathogenic	10	3.1	58	F	22.6	9	-	[23]
14	intron 17	c.2547+1G>A	p.?	splicing	Pathogenic	11.1	8.9	66	M	24.8	18	-	[24]

**Table 3:** Clinical and phenotypic data on Hungarian patients with *LDLR* variants and detailed summary of *LDLR* variants—patients with compound heterozygous *LDLR* variants.

Patient ID	Exon	Variant at cDNA Level	Variant at Protein Level	Variant Type	ACMG Classification	TC (mmol/L)	LDL-C (mmol/L)	Age	Gender	BMI	Dutch Score	Xanthoma, xanthelasma	Reference
12	11	c.1618G>A	p.Ala540Thr	missense	Pathogenic	21.7	n/a	26	F	29.36	20	+	[19]
	7	c.941-190_c.1061-270del	?	large deletion	Pathogenic								This study
13	4	c.337G>T	p.Glu113*	nonsense	Pathogenic	23.7	n/a	38	M	20.15	20	+	[11]
	11	c.1618G>A	p.Ala540Thr	missense	Pathogenic								[19]
28	6	c.862G>A	p.Glu288Lys	missense	Pathogenic	10.4	10.8	13	M	29.4	17	-	[25]
	15	c.2167delG	p.Glu723Arg fs*7	frameshift	Pathogenic								[18]

**Table 4:** Clinical and phenotypic data in Hungarian patients with *LDLR* variants and detailed summary of *LDLR* variants—patients with *LDLR* CNVs.

Patient ID	Exon	Variant Type	ACMG Classification	TC (mmol/L)	LDL-C (mmol/L)	Age	Gender	BMI	Dutch Score	Xanthoma, xanthelasma	Reference
38	exon 4–8	duplication	Pathogenic	16.6	9.8	69	F	25.7	24	+	[26]
33	exon 3–8	deletion	Pathogenic	n/a	n/a	74	F	40.4	10	-	[27]

## Data Availability

The data presented in this study are available on request from the corresponding author.

## Supplementary Materials

The following are available online at: https://www.mdpi.com/article/10.3390/genes13010153/s1, Table S1. *LDLR* amplification and sequencing primers, Table S2. Important clinical and phenotypic data in Hungarian patients with *LDLR* alterations and detailed summary of *LDLR* variants—Family members.
